# Using Beatboxing for Creative Rehabilitation After Laryngectomy: Experiences From a Public Engagement Project

**DOI:** 10.3389/fpsyg.2019.02854

**Published:** 2020-01-14

**Authors:** Thomas Moors, Sanjeev Silva, Donatella Maraschin, David Young, John M. Quinn, John de Carpentier, Johan Allouche, Evangelos Himonides

**Affiliations:** ^1^Shout at Cancer, London, United Kingdom; ^2^School of Arts and Creative Industries, London South Bank University, London, United Kingdom; ^3^School of Science and Engineering, University of Dundee, Dundee, United Kingdom; ^4^First Faculty of Medicine, Institute of Hygiene and Epidemiology, Charles University, Prague, Czechia; ^5^Royal Preston Hospital, Preston, United Kingdom; ^6^Hôpital Saint-Pierre, Brussels, Belgium; ^7^UCL Institute of Education, University College London, London, United Kingdom

**Keywords:** head and neck cancer, throat cancer, voice rehabilitation, laryngectomy, beatboxing

## Abstract

Laryngectomy is the surgical removal of the larynx (voice box), usually performed in patients with advanced stages of throat cancer. The psychosocial impact of losing the voice is significant, affecting a person’s professional and social life in a devastating way, and a proportion of this patient group subsequently must overcome depression (22–30%) and social isolation (40%). The profound changes to anatomical structures involved in voicing and articulation, as a result of surgery, radiotherapy or chemotherapy (separately or in combination with one another), introduce challenges faced in speech rehabilitation and voice production that complicate social reintegration and quality of life. After laryngectomy, breathing, voicing, articulation and tongue movement are major components in restoring communication. Regular exercise of the chest, neck and oropharyngeal muscles, in particular, is important in controlling these components and keeping the involved structures supple. It is, however, a difficult task for a speech therapist to keep the patient engaged and motivated to practice these exercises. We have adopted a multidisciplinary approach to explore the use of basic beatboxing techniques to create a wide variety of exercises that are seen as fun and interactive and that maximize the use of the structures important in alaryngeal phonation. We herein report on our empirical work in developing patients’ skills, particularly relating to voiced and unvoiced consonants to improve intelligibility. In collaboration with a professional beatboxing performer, we produced instructional online video materials to support patients working on their own and/or with support from speech therapists. Although the present paper is focused predominantly on introducing the structure of the conducted workshops, the rationale for their design and the final public engagement performance, we also include feedback from participants to commence the critical discourse about whether this type of activity could lead to systematic underlying research and robustly assessed interventions in the future. Based on this exploratory work, we conclude that the innovative approach that we employed was found to be engaging, useful, informative and motivating. We conclude by offering our views regarding the limitations of our work and the implications for future empirical research.

## Introduction

Head and neck cancers (HNC) are a burgeoning public health burden worldwide, causing significant mortality and morbidity despite clinical advances enabling early diagnosis and treatment (Gupta et al., 2016, 2017). The estimated incidence rates of HNC are shifting toward predominance in the less and least developed regions of the world, where health-care offerings may be inadequately equipped to diagnose and appropriately treat HNC and, thus, health-care outcomes may be much worse as a result (Gupta et al., 2016). Surgical intervention for HNC requires significant expertise and can often have a serious impact on the quality of daily life; therefore, exploring novel support strategies and activities as part of a holistic approach in rehabilitation might ensure improved quality of life among those affected.

## Background

### Epidemiology

Head and neck cancers include cancer of the lips, oropharynx, hypopharynx, pharynx, major salivary glands, larynx and sinuses according to the WHO classification of head and neck tumors and series on histological and genetic typing of human tumors (Gatta and Botta, 2017; Wright and Vered, 2017). HNC are considered a rare cancer with reported annual incidence rates of less than six per 100,000 individuals and a prevalence of less than five per 10,000 individuals in some populations (Gatta and Botta, 2017).

A large proportion (35%) of cases of HNC is laryngeal cancer (Cancer Research United Kingdom, 2019). A laryngectomy is only suggested in the advanced stages of throat cancer in an effort to mitigate metastasis and mortality in this patient group. In developed countries, the number of laryngectomies performed is low because of early presentation and ease of access to health care involving combinations of radiotherapy and chemotherapy. In the United Kingdom (UK), for example, each year there are approximately 500–600 laryngectomies performed, with 542 conducted in 2016–2017 (NHS, 2018). In the developing world, however, many laryngeal cancers are diagnosed at more advanced stages and require total laryngectomy (Staffieri et al., 2006).

Patients with HNC, in common with other ‘rare cancers’, face difficulties in diagnosis, treatment planning, power of research and organization of the disease management approach (Gatta and Botta, 2017). In developing countries, this problem may be worse due to economic reasons limiting both access to health-care provision that might allow early diagnosis and perhaps lead to non-surgical therapies, thus avoiding the need for laryngectomy (Staffieri et al., 2006; Stevens and Huys, 2017).

### Laryngectomy

Laryngectomy is usually performed in patients at later stages of throat cancer. Cancer definitions from the new WHO update divide tumors of the oral cavity and oropharynx into separate chapters, classify SCCs of the oropharynx on the basis of HPV status, abandon the practice of histologic grading for oropharyngeal SCCs that are HPV-positive, recognize small cell carcinoma of the oropharynx, and combine polymorphous low-grade adenocarcinoma and cribriform adenocarcinoma of the tongue and minor salivary glands under the single term ‘polymorphous adenocarcinoma’ (Westra and Lewis, 2017).

In the presurgical situation, air is expelled from the lungs and passes from the trachea (the windpipe) through the larynx, where vibrations of the mucosa over the vocal folds (the glottis) create a mucosal wave; this is known as the *fundamental frequency*. In contrast, everything else above the laryngeal assembly that participates in the shaping, amplification, dampening and branding of the sound produced by the glottis is customarily called *the vocal tract* (including the soft tissue, cartilage, nasal cavity, tongue, teeth and lips; Welch et al., 2019).

**FIGURE 1:**
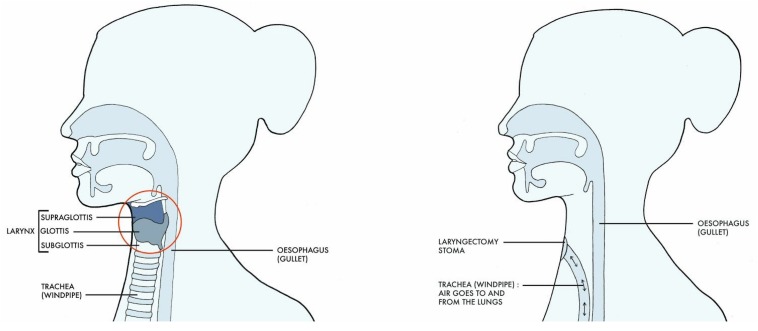
Anatomical changes seen before and after laryngectomy (Drawing Claire Holmes).

When performing a laryngectomy, the surgeon preserves as much pharyngeal mucosa as possible to limit the defect created and make the reconstruction of the neopharynx easier, avoid or minimize flap reconstruction, and facilitate voice recovery (Elmiyeh et al., 2010). The trachea is detached from the larynx and brought forward to be attached to the skin, creating a stoma (opening) in the front of the neck. It is important to note that, upon laryngectomy, the patients’ airway is not directly connected with the mouth anymore: instead, they are breathing in and out of the neck (Elmiyeh et al., 2010; Figure 1).

After removal of the larynx, a PE segment is reconstructed with a resonating pharyngeal segment above it (Elmiyeh et al., 2010). The resonating segment will act as the main source of vibration of the air expelled from the lungs and diverted into this segment, resulting in sound production; it is therefore called the neoglottis (Elmiyeh et al., 2010).

The procedure is, in effect, a substantial amputation of the anatomy responsible for sound production. It affects both the afferent and efferent pathways for voice production and control. The whole speech production process and co-ordination, often including hand movements, need to be relearned or adjusted depending on which type of voice restoration has been employed.

There are four different ways to restore speech, with TE voice among them nowadays considered to be the gold standard (Jongmans et al., 2006; Elmiyeh et al., 2010; Bohnenkamp et al., 2011).

### TE Voice

**FIGURE 2:**
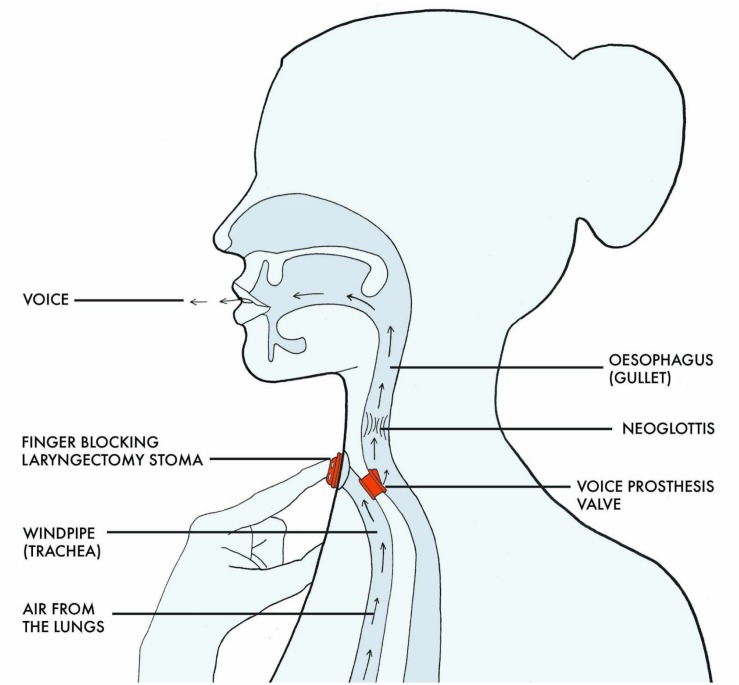
Airflow and voicing in TE speech (Drawing, Claire Holmes).

In this approach, a surgical puncture allows the placement of a replaceable unidirectional valve that diverts air from the trachea into the esophagus (Figure 2).

When the stoma is occluded, the air has to escape and is therefore pushed through the valve, causing vibration of the neoglottis. Because of the small diameter of this valve, it takes approximately 7.5 times more pressure to achieve voicing when compared with normal laryngeal phonation (Bohnenkamp et al., 2011).

To recommence speaking after laryngectomy, a new co-ordination strategy is required to prepare and control breathing and for manually occluding the stoma at precisely the right moment so that sufficient pressure can build up to open the valve and divert air through the valve into the neopharynx, where it forms a vibrating column of air as it passes upwards through the neo-glottis. This column of vibrating air can be articulated into sounds and speech similar to the way described earlier (Bohnenkamp et al., 2011).

Overall, this approach produces a more natural sound, requires greater power from the lungs, facilitates more volume control (van As et al., 1998; Jongmans et al., 2006; Kaye et al., 2017), makes it easier to differentiate between voiced and unvoiced consonants (Jongmans et al., 2006), is expensive and necessitates that the valve be replaced every 3–6 months (Staffieri et al., 2006), and offers patients a higher degree of voice satisfaction (Kaye et al., 2017).

### Electrolarynx (EL) Voice

With this solution, there is no valve connecting the trachea with the neopharynx; instead, an artificial vibrating device is held against the user’s throat or cheek. When switched on, controlled by its user’s thumb, the sound vibration is transmitted to the oral cavity, where it is articulated into speech (Elmiyeh et al., 2010; Kaye et al., 2017).

This approach produces a monotonous, electronic sound; facilitates the speaking of unlimited words; is easy to learn; requires an expensive first purchase but subsequent maintenance is cheap; and makes it difficult to differentiate voiced from unvoiced consonants, depending on hand coordination (Kaye et al., 2017).

### Esophageal (E) Voice

E speakers don’t have a valve connecting the trachea with the esophagus. Instead, air is drawn into the upper esophagus and then released into the mouth, producing vibrations in the pharyngeal and PE wall that generates a sound (Elmiyeh et al., 2010).

Here, the sounds, volume and number of words are limited; the sound produced is monotonous; the technique is harder to learn; there is no extra cost or need for regular valve replacements; and it is difficult to differentiate voiced from unvoiced consonants (Kaye et al., 2017).

### Mouthing or Lip Speech

When all other options fail or are not available, patients can over-articulate and make limited use of sounds solely using their mouths. With this approach, no sound is produced, there is difficulty differentiating voiced and unvoiced consonants, intelligibility is limited and there is no need for medical devices.

One of the difficulties experienced in speech after laryngectomy, in addition to the issues of volume control and pitch range, is the differentiation between voiced and unvoiced consonants (Jongmans et al., 2006). The variation between these consonants simply depends on whether or not the voice is used to support the articulation. TE phonation demands a controlled expiration through the valve that causes the PE segment to vibrate. For E speech, this is even more difficult, as the phonation comes from swallowed air, while, in the context of EL voice, it depends on the digital control of the device. Therefore for both E and EL phonation, success comes down to the combination of lip speech and their voicing techniques. Lip speech is hardly practiced in the West, but we assume that it is more frequent in developing countries where patients face difficulty in accessing facilities and medical devices (Staffieri et al., 2006). Nevertheless, evidence suggests that it will add to the clarity of both E and EL voice.

The psychosocial impact of losing the voice is significant, affecting a person’s professional and social life in a devastating way (Dooks et al., 2012; Keszte et al., 2013). A high percentage of this group suffers from social withdrawal (40%; Danker et al., 2010) and depression (22–30%; Bussian et al., 2010; Danker et al., 2010; Dooks et al., 2012; Keszte et al., 2013; Perry et al., 2015).

### Speech Production

Normal speech (and singing) requires the involvement of more than 100 muscles. It is remarkable that this process takes place entirely within the body without visual control over movement (Kleber et al., 2010).

The interaction between the activity of the vocal folds, larynx, respiration and articulators is fine-tuned (Dejonckere and Lebacq, 1981) and performed at a fast rate, necessitating the presence of a control system that mainly depends on an intrinsic reflex system (Abo-El-Enein and Wyke, 1966).

This complex process is thought to become automatic in speech once development is complete (Smith and Zelaznik, 2004). It is obvious that a laryngectomy or the removal of the anatomical part responsible for initiating the vibration that results in voicing affects this vocal motor system.

Cohen et al. (1991) showed that lesions, like amputations, cause a neuroplastic reorganization of motor outputs in the brain targeting the muscles proximal to the injury, allowing for a rapid reallocation of the available neural networking. We can expect a similar response in the affected vocal motor system after laryngectomy because of the suggested specific cortical area for the larynx and articulators (Kleber et al., 2010), even though the laryngeal and orofacial muscle fibers are distinct from peripheral muscles (Kent, 2004).

It is suggested that regular practice with great attention to auditory and kinesthetic feedback (e.g., from laryngeal mechanoreceptors) for vocal control helps voice professionals optimize the co-ordination of the vocal motor system, including the articulators and larynx (Sundberg, 1987; Mürbe et al., 2004).

Kleber et al. (2010) also suggest that vocal training increases the involvement of implicit memories of movement control, while Mürbe et al. (2004) postulated that the auditory feedback is most important in the early stages of vocal training, with a fundamental role in pitch control, and that the kinesthetic feedback circuit seems to be particularly improved (e.g., in classical singing) after years of training (Mürbe et al., 2004).

Therefore we suggest that, during the (initial) relearning phases for controlling the new vocal instrument in order to speak, patients who underwent laryngectomy need to be supported whilst exploring their voices and practicing phonation effectively and systematically. Any effective means to foster, maintain, and/or increase the motivation for patients to practice and try to improve would be welcome.

### Beatboxing

At its base nature, beatboxing is the art of vocal percussion; more recently, it has been linked to what is now described as ‘hip-hop’ culture and is popular amongst younger audiences and artists (Stowell and Plumbley, 2008). Beatboxing employs multiple beat modalities, including vocal instruments, to produce both rhythmic and melodic sounds. These sounds are often perceived as overlapping (occurring in synchrony) in time. The majority of beatboxing sounds imitate percussion instruments like drums and cymbals yet are also seen as similar to speech sounds and can be described using symbols from the International Phonetic Alphabet (IPA; Stowell and Plumbley, 2008; Stowell and Plumbley, 2010; Proctor et al., 2013)^1^ or with the use of characters from a standard English computer keyboard as in the SBN (Splinter and TyTe, 2002).

Despite this similarity with sounds used in speech, beatboxers explore their instruments continually and have been ‘inventing’ and introducing novel sounds that are non-native to them (Proctor et al., 2013) or even extralinguistic (Proctor et al., 2013; de Torcy et al., 2014).

Beatboxing is inexpensive, as no purchase of instruments or technical equipment is required to start learning the basics. Beatboxing is also presented as a pluralistic and democratic artform (Himonides et al., 2018) where ‘every sound is valid.’ We felt that it would be worth investigating whether this inexpensive and easily accessible activity could be of use in speech pathology and, particularly, in rehabilitation after laryngectomy.

### Why Beatboxing and Laryngectomy?

Proctor et al. (2013) showed that beatboxers, like other voice professionals, display an increase in the sensorimotor areas specific for voicing but that this fine-tuned control is ‘exploited’ to obtain a musical effect.

Due to the surgical changes in anatomy after laryngectomy, other interesting and potentially beneficial aspects of beatboxing include the skill of detaching laryngeal from pharyngeal activity (de Torcy et al., 2014) using the hypopharynx as an individual resonator (Kitamura et al., 2005) and the ability to create plosive sounds with a closed glottis independent of the airflow used for breathing support (de Torcy et al., 2014). An analysis of imaging (Proctor et al., 2013) showed a diversity existed in tongue movement, supporting the possible benefits of beatboxing techniques in promoting suppleness and linking articulation to breathing control and voicing.

Overall, all of these factors support the usefulness of beatboxing in TE, EL, E and lip speech. The rhythmic, playful and explorative approach makes it a useful tool to motivate people to practice co-ordination in voicing and improve intelligibility.

Here, we used beatboxing to explore the alaryngeal voice, breathing control and vocal pitch, paying particular attention to unvoiced and voiced sounds. An additional reason for exploring the use of beatboxing was because it is perceived to be a fun activity, is simple and cheap to conduct, and can be readily adapted for online participation, thus improving accessibility.

## Materials and Methods

This project involved Shout at Cancer (a non-profit organization specializing in post-laryngectomy voice), Marv Radio (a beatboxer), UCL music education researchers/facilitators, a group of cancer survivors with laryngectomy coming from across the United Kingdom, local East London youth, and an audience (for the final public performance) that involved families and guests from across London and the United Kingdom.

There were nine laryngectomy patients, including six males and three females, with a mean age of 65 years. Seven used TE voice, one voiced with an EL, and one relied on lip speech or mouthing.

Inclusion criteria were total laryngectomy using TE, E, EL or lip speech. There were no exclusion criteria.

The present study did not require research ethics approval as confirmed by the joint Medical Research Council (MRC) and United Kingdom National Health Service (NHS) Health Research Authority online ethics assessment tool^2^. Nevertheless, written informed consent was obtained from all participants and, in the case of the young performers, written informed consent was obtained from their legal guardians.

### Workshops

We organized five workshops, each lasting 2 h, that were held weekly. The defined goals of the workshops were:

(1) To engage a vulnerable group of individuals in collaborative music-making using novel techniques (i.e., beatboxing),
(2) To engage a wider group of local youth in East London in artistic expression and collaboration with cancer patients, and
(3) To engage a wider public audience in an open showcase of masterclass outcomes/concert to explore the use of beatboxing techniques in laryngectomy.

During the workshops, we explored whether beatboxing techniques are applicable in speech rehabilitation after laryngectomy. Patients, clinicians and speech-language pathologists were invited to participate with the beatboxer in developing vocal and breathing skills. We focused on unvoiced and voiced consonants in lip speech, EL and TE voice. We followed the basic beatboxing sounds described in Tables 1, 2. We approached the exploration of the consonants, used in the English language, as beatboxing sounds (Table 3). Participants practiced the sounds separately at first and then in different rhythms and combinations to refine and improve the hand, breathing and voicing co-ordination.

**TABLE 1:** Musical classification and the phonetic description of the basic beatboxing sounds used during the workshops based on the description used in prior research (Proctor et al., 2013; IPA, 2015), the SBN (Splinter and TyTe, 2002) and the phonological description of consonants (O’Grady et al., 2017).

**Name/picture**	**Description**	**SBN**	**IPA**
Rimshot 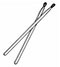	Imitation of the sound of hitting the drumstick against the rim of the drum or like two drumsticks hit against each other. The sound is achieved by pronouncing the **k**, a voiceless velar stop or plosive.	{k}	[k']
Classic kick 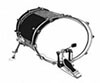	Soft and low pitch sound, an imitation of the big drum on the drum set. The sound is achieved by pronouncing the letter **b**, a voiced bilabial stop or plosive.	{b}	[pʼɪ̥]
Basic or Closed hi-hat 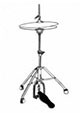	A high-pitched and prolonged sound imitation of the hi-hat, as it is resonating more in the open position. The sound is achieved by adding a prolonged **s** to the **t**. Made by starting with a voiceless alveolar stop or plosive (t) and adding a prolonged voiceless alveolar fricative (s).	{t}	[t̻͡s t̻^¬^]
Dry Kick 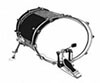	A low pitched sound imitation of the big drum on the drum set, like the earlier described classic kick. The sound this time, however, is achieved by pronouncing a **d**, a voiced alveolar stop or plosive.	{d}	[dɪ̥]
Open hi- hat 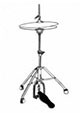	A high pitch and prolonged sound imitation of the hi-hat, as it is resonating more in the open position. The sound is achieved by adding a prolonged **s** to the **t**. Starting with a voiceless alveolar stop or plosive (t) and adding a prolonged voiceless alveolar fricative (s).	{ts}	[t̻͡͡sː]
Classic snare 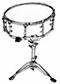	The snare drum owes its typical sound to the metal strings (snare) mounted on its underside. When the drum is hit, the snare starts vibrating over the drum skin below. The sound is achieved by pronouncing a **p**, a voiceless bilabial stop, or plosive, and adding a prolonged **f**, a voiceless labiodental fricative, to it.	{pf}	[p͡fʼːú̥]
Cymbals 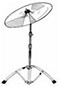	The cymbal is loud, high-pitched, and has a lot of resonation. The sound is achieved by starting with a forced **t**, voiceless alveolar stop or plosive and adding a well-supported **‘sh’** sound, a voiceless alveolo-palatal fricative, to it.	{T}	[tɕːʷ]

**TABLE 2:** Additional sounds (IPA, 2015).

**Name**	**Sound description**	**IPA**
Wood block 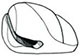	A woodblock is a percussion instrument that has a warm and hollow sound; it comes in different sizes, each with a different pitch. The sound is achieved by a tongue click, apical (post)alveolar click and changes in the shape of the mouth cavity to change pitch.	[!]
Whip or Slapstick 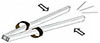	The whip, slapstick, or clapper creates a cracking noise. The sound is achieved by a tongue click, a laminal postalveolar click and a simultaneous mouth opening.	[ǂ]

**TABLE 3:** Voiced and unvoiced consonants described anatomically and according to the manners of articulation (O’Grady et al., 2017).

**Manner of articulation**		**Unvoiced/voiced**	**Anatomical placement of the articulated consonant**					
			**Bilabial**	**Labiodental**	**Interdental**	**Alveolar**	**Palatal**	**Velar**
Obstruent	Stop	Unvoiced	p			t		k
		Voiced	b			d		g
	Fricative	Unvoiced		f	θ	s	∫	
		Voiced		v	ð	z	ʒ	
	Affricate	Unvoiced					ʧ	
		Voiced					ʤ	
sonorant	Nasal	Voiced	m			n		η
	Liquids	Voiced				I	ʴ	
	Glides	Voiced	w			j		

*Note that the glottal sounds have been left out because of the level of difficulty for the laryngectomy patient.*

In the last two sessions, we included local youth, specifically four boys and two girls between six and 13 years old, who were introduced to basic beatboxing. They practiced together with the patients after an introduction and explanatory talks about laryngectomy. We prepared the songs of the program for the concert 3 weeks later. They were encouraged to interact with the patients and to ask questions.

**FIGURE 3:**
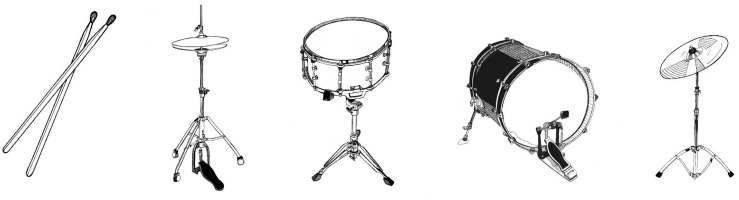
From left to right: Rimshot, Hi-hat, snare drum, kick drum, and cymbal. When the picture is displayed, the patient is invited to imitate his/her interpretation of the sound made by the instrument. Thanks to a visual input only, the patient is freer to explore his or her expression without having been influenced by someone else’s performance.

At first, we introduced three basic beatboxing sounds (see Figure 3): the classic kick drum ({b}, [pʼɪ̥]), the basic or closed hi-hat ({t}, [t̻͡s t̻^¬^]) and the rimshot ({k} [k']). We started to work on each sound separately to focus on pronunciation, controlling volume and tempo. To practice the control of volume, we pronounced the same sound at different levels of loudness, from soft to loud and then at random (e.g., for the kick drum: b,b,b; B,B,B; B,B,B; or bBb).

We worked on the co-ordination of breathing and voicing by repeating the same sound in different tempos, increasing the speed of pronouncing the same sound and progressing from slowly to as fast as possible. During the exercise, it was important to pronounce the sound properly.

For example: b,  b,   b    b,b,b     b,b,b

We combined both exercises to work on a better control of speed and volume at the same time For example: b, B, B, bbb, B, B, bbb.

The next step, to make it more playful and to help participants understand how easily these exercises can be built into their daily routine, we exercised with rhythms from familiar dance styles or famous songs (e.g., waltz, samba, tango, salsa, ‘We Will Rock You’ by Queen). The integration of these exercises into music facilitated practicing individually.

The workshops were structured in a repetitive way with a variety of actions with an increasing level of difficulty.

The next step focused on developing the participants’ control of the volume of a sequence of different sounds, allowing them to link breathing and voicing control to a limited set of different positioning of the articulators and paying careful attention to its pronunciation.

For example: b,t,k   B,T,K   **B,T,K** or b**T**k.

Again, we continued by increasing the speed of the pronounced sounds.

For example: b,    t,    k    b,t,k b,t,k,

We ended up going really fast and practiced using rhythms from familiar dance styles or famous songs. The changes in tempo and in rhythm add a gradient of fun to the exercise; they also make it a cognitive exercise as it demands more concentration.

Once the participants understood basic beatboxing—that is, the idea of repeating sounds in different levels of volume and rhythms—we started to introduce more sounds and helped them interact more with the beatboxer and each other.

The beatboxer demonstrated challenging combinations that they had to repeat all together, in pairs or individually.

Several times during the workshops, we conducted battles where the beatboxer challenged the participants or the participants had to ‘provoke’ each other with difficult combinations for the other to repeat.

For example: b, t, t, K, T    b, t,t,t,t k, K, k, B.

Near the end of each session, we spent time trying to come up with new sounds. Initially, this idea appeared difficult to understand and was met with some hesitation. To support the participants with the challenge, we invited them to imitate different sounds that we are likely to be familiar with, such as a dog barking, a helicopter, a car passing by, a car braking or honking, a phone ringing or a bee buzzing.

When the group felt more confident, we let the participants and the children challenge others in the group with different sounds they had invented. This was a playful way to improve the cohesion of the group and help each other explore sounds.

By the end of the series of workshops, we had built up a repertoire of varying beats and vocal tricks and integrated these in the selection of songs for our performance. In one of the songs, we imitated the loud world we live in, building up the chaos gradually with the whole group together. We then stopped abruptly and made the crowd reflect: imagine struggling with your voice, living in our loud and fast world.

The main beatboxing sounds we included in the further workshops and the music we prepared for the concert were cymbals ({T}, [tɕːʷ]), basic or closed hi-hat ({t}, [t̻͡s t̻^¬^]), open hi-hat ({ts}, [t̻͡͡sː]), rimshot ({k}, [k']), classic snare ({pf}, [p͡fʼːú̥]), classic kick drum ({b}, [pʼɪ̥]) and dry kick drum ({d}, [dɪ̥]).

**FIGURE 4:**
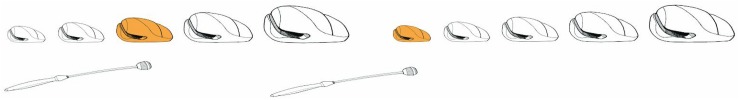
Woodblocks are displayed from left to right in decreasing pitch or increasing size. The highlighted size of the woodblock changes each time the picture is displayed, inviting the participant to explore different (apical alveolar) clicking sounds and different pitches achieved by adjusting the tongue movement and mouth opening.

**FIGURE 5:**
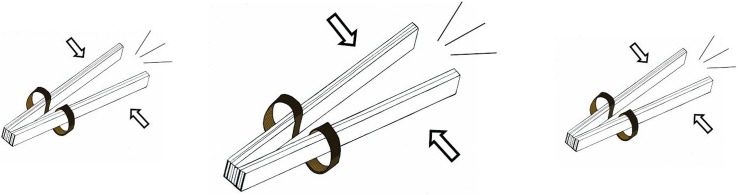
A whip or slapstick. These pictures invite the participant to explore a laminal postalveolar click. By changing the size of the displayed picture, they must react accordingly with volume control. This way, they learn to control the tongue pressure and release in combination with mouth opening in order to influence the volume of the sound. Such helps to practice using the involved articulators, including mainly the tongue.

Tongue-click sounds were a way to do warm-up exercises for the jaw and the tongue (e.g., whip or slapstick [ǂ] or wood block [!]) (Figures 4, 5). These also represent an easier way to practice rhythms within a group, as they do not require co-ordination among breathing, hand movement, voicing and articulation. Furthermore, it showed to the patients that sound can be created without voicing.

### Lip Speech or Mouthing

To explain how beatboxing could possibly make lip speech or mouthing more clear, we focused on tongue clicks at first. The beatboxer demonstrated how changing the shape and opening of the mouth influences the pitch of the sound. We then practiced pitch control of the clicking sounds in the group and individually. The next step was to shape the vocal tract to obtain the vowels A, E, I, O, and U in combination with the clicking sounds. We repeated the vowels obtained with clicking sounds, changing the volume, rhythm and pitch.

After the group felt more confident in being creative enough to give shape to vowels without using voice, we explained what the difference is between voiced and unvoiced consonants. Table 3 shows the protocol/set of sounds that we tried to follow systematically. We focused on tackling the production of the paired sounds t and d, p and b, f and v, s and z, and k and g because these were close to the basic beatboxing sounds we had covered already.

For each pair, we repeated each one four times (e.g., p p p p b b b b, p p p p b b b b). To make it slightly harder, we included variations in tempo and volume. Then, we encouraged the participants to pronounce the voiced and non-voiced paired consonants alternatively (e.g., p b p b p b p b), again continuing with changing the required volume and tempo. We finished these series of exercises by combining all the sounds randomly where the group had to imitate the voice coach or each other when we divided them up in pairs to practice.

At this point, the participants were paying more attention to the co-ordination in using or not using voice in articulation.

We then explored in a similar way as above the more subtle paired consonants ð, ʃ and ʒ, and ʧ and ʤ. The nasal sounds m, n and η are difficult to pronounce in mouthing or lip speech and we did not find beatboxing sounds to make these consonants easier to differentiate.

We approached the consonant L in a similar way as the tongue clicks, changing the pitch of the sound by adjusting the mouth opening. The exercise here was to make the sound of pouring water from a bottle in a glass, in a slow or fast way.

### TE Speech

The exercises for TE speech focused on linking breathing to hand co-ordination, phonation and articulation and establishing a clear distinction between the voiced and unvoiced consonants.

We aimed to begin each workshop with breathing exercises as a warm-up, paying a lot of attention to control the expiration through the speech valve and out of the mouth without causing the neoglottis to vibrate or phonate.

We encouraged the participants to prolong the unvoiced consonants f and s for 5 s, building up to 10 s. The next step was to steadily grow louder or the other way around. Following this, they were helped to pronounce an f or s sound five times, each lasting approximately for a second, followed by a 1-s pause. Once they were able to control the f and s sounds, they were asked to flow over from a prolonged f into a v sound or an s into a z. Then, we started to practice series of five times repeating f and v alternatively (e.g., f v f v f v f v f v or s z s z s z s z s z).

These exercises seemed to help the participants focus on voiced and unvoiced sounds and, from then on, we systematically followed the consonants presented in Table 3. We tackled the following paired sounds—t and d, p and b, f and v, s and z, and k and g—because they are close to the basic beatboxing sounds we had covered already.

Next, we made them pronounce the voiced and unvoiced paired consonants alternatively (e.g., p b p b p b p b), again continuing with changing the required volume and tempo. We finished these series of exercises by combining all of the sounds randomly in a scenario where the group had to imitate the voice coach or each other when we divided them into pairs to practice.

We then explored in a similar way as above the more subtle paired consonants θ and ð, ʃ and ʒ, and ʧ and ʤ. Unlike in lip speech or mouthing, the nasal sounds m, n and η are easy to pronounce in TE speech. In fact, we used these sounds to work on resonance and made the group imitate car or bike engines that were accelerating, slowing down, or hitting their brakes.

The advantage of TE speech, unlike the other voice restoration possibilities, is the airflow that supports voicing. An interesting exercise to control airflow with or without voicing is rolling the r sound. To make it engaging, we imitated growling dogs.

### The Concert

These workshops culminated in a public performance, the world premiere of Beatboxing Without a Voice, at the Olympic Village, Stratford, East London on 8 April 2017. This concert was an interactive session involving local people and families across London, the patients, an opera singer and the beatboxer. The research team also offered brief explanatory talks presenting the layered impact of throat cancer and laryngectomy. There were over 130 people in the audience (this included confirmed bookings as well as Olympic Village visitors who tagged along without prior registration).

Those at the public performance were invited to provide feedback not only about their experience but also about the knowledge that they gained/acquired regarding throat cancer.

## Results

### Pictures

We translated the basic sounds of beatboxing (Tables 1, 2), into pictures and invited artist Claire Holmes to create bespoke graphics for our learning materials (Figure 3).

### Video

We created a series of video exercises in which the beatboxer first demonstrates the basic beatboxing sounds one by one, followed by a variety of different combinations of these basic beatboxing sounds in different levels of difficulty and tempo. Participants are invited to repeat each exercise.

### TE Consonants

Thanks to breathing control, it is possible to make a difference between voiced and unvoiced consonants in the TE voice. We had the patients practice different rhythms and sound combinations in which there is a change from voiced to unvoiced consonants (Table 2).

### Lip Speech Consonants

We explored how to make the differences between voiced and unvoiced consonants used in English more clear by adding sounds supporting the voiced consonants (Table 3; also, a supporting video on the Shout at Cancer website^3^ is available).

### Electrolarynx

We provided an EL to our beatboxer, who explored and demonstrated the beatboxing possibilities with the device.

With our laryngectomy participants, we worked on the combination of lip speech techniques to obtain unvoiced consonants without the use of the electrolarynx and a smooth co-ordination of the use of the device to obtain vowels and voiced consonants. These demand a high amount of attention, initially is frustrating to use, and is hard to maintain^4^.

### Workshop With Local Youth

The workshop allowed the patients to be more comfortable and to explore sounds without judgement from outside the group. It also helped us in the preparation of the patients and to get the youth familiarized with the aftereffects of laryngectomy. We included the young participants in talks about laryngectomy during the performance.

### Working With Voice Professionals

For some of the voice professionals, it was the first time they had been involved in a beatboxing program. Although focused on the alaryngeal voice, it was interesting for the health-care professional to explore the voice in a different way, determine overlapping skills and be able to explain sounds in a different way. The team reported to have benefited from the techniques learned in the project and will be able to implement these in their own clinical or client-based activities.

### Beatboxer

The beatboxing artist involved in the project faced the incredibly challenging task of having to learn to take other people’s physical constraints and limitations into account and also being required to form an understanding about the pathology and social impact on patients after laryngectomy. He worked hard in trying to explore a voice with so many restrictions. This was an incredibly challenging process for a freestyle artist, and the research team witnessed a professional with incredible talent. The interaction with both patients and health-care professionals was challenging, and the learning curve was steep and required continual adjustment and critical thinking.

### Final Performance and Feedback

The project exceeded the aims set out in the initial proposal. Beatboxing after laryngectomy had an impact at several levels, including on both the individuals and the partners involved and potentially on future research.

However, this type of activity was not viewed enthusiastically by all laryngectomees. One participant was particularly negative about beatboxing as an artform and reported that they did not enjoy the workshops, the music or working with the beatboxing expert. Somewhat paradoxically, though, even that particular participant reported that the umbrella of activities leading to the final public engagement concert seemed to offer some benefits for developing breathing control as well as for exercising the different structures for alaryngeal phonation. This was particularly due to beatboxing’s strong reliance on rhythmic precision and adhering to strict rhythmical patterns.

Although the present work was primarily centred on public engagement and was not intended to form a clinical research study or intervention study, the team nonetheless decided to record some feedback from the participants and the participating audience. This was seen as essential to gauge the potential or value for similar work to play a key role in a future, systematically researched project.

Throughout the span of this work, the laryngectomees, the core team, the artist, and the collaborating speech and language pathologists/therapists worked in synergy to tweak the beatboxing exercises/tasks to a level where there was a good balance achieved between task-appropriateness for the patients and artistic value for a beatboxing performance. This proved to be a very meaningful exploratory process where not only key challenges but also useful methods were identified.

All participant laryngectomees were invited to offer feedback about their beatboxing experience using SMS messaging (for convenience) and/or email. Participants were asked to rate the extent to which they agreed or disagreed with the following six statements:

(1) I enjoyed participating in the project,
(2) I benefited psychologically from participating in the project,
(3) My voice production ability has benefited from participating in the project,
(4) I felt more confident about myself after participating in the project,
(5) I would recommend beatboxing to other laryngectomees,
(6) I would participate in a beatboxing project again in the future.

All responses appeared to be positive but not overwhelmingly so (Table 4). Nevertheless, as hinted above, only one laryngectomee appeared to have an overall negative view about their participation in the project; all other respondents offered ratings with a strong sense of positivity (average score = 6.2 points, standard deviation = 1.01 points).

**TABLE 4:** Laryngectomees’ short evaluation of their beatboxing experiences.

**Participant**	**Q1**	**Q2**	**Q3**	**Q4**	**Q5**	**Q6**	**average**
p1	7	7	7	7	7	7	7.0
p2	7	7	7	7	7	7	7.0
p3	7	7	7	7	7	7	7.0
p4	6	4	3	5	5	5	4.7
p5	7	6	7	6	7	7	6.7
p6	7	4	6	4	5	4	5.0
p7	3	3	3	3	3	3	3.0
p8	7	3	7	5	–	7	5.8
Average	6.4	5.1	5.9	5.5	5.9	5.9	5.8

Out of the 130+ final concert participants, 58 individuals offered feedback on an online survey instrument, a link to which was made available post-concert using registered participants’ email addresses. Participants were allowed to offer ratings about the extent to which they agreed or disagreed with three statements. They were also offered the chance to provide free text feedback in a dedicated textbox. Ratings were performed on a seven-point Likert-type scale, and the available scores ranged from one point (completely disagree) to seven points (completely agree), with four points denoting neutrality (neither agree nor disagree). The three statements that participants were invited to rate were: ‘I enjoyed participating in this event,’ ‘ I feel that my understanding about laryngectomy is greater because of this event’ and ‘I would like to attend a similar event in the future.’ Responses were overwhelmingly positive, therefore negating the need for the identification of commonality or diversity in response between different age or sex groups. Table 5 summarizes participants’ responses to the three statements.

**TABLE 5:** Final performance participants’ online evaluation.

**Question**	** Number of responses per scale item**							**Mean rating**	**Total**
	**1**	**2**	**3**	**4**	**5**	**6**	**7**		
I enjoyed participating in this event	0	0	0	0	0	8	50	6.86	58
I feel that my understanding about laryngectomy is greater because of this event	0	0	0	1	7	9	41	6.55	58
I would like to attend a similar event in the future	0	0	0	2	3	10	43	6.62	58

In addition to the rating of the three statements, 18 participants offered further comments in the available textbox. In line with the inordinate positivity shown in the ratings, participants offered optimistic commentaries. Some examples are as follows:

• It was a great experience. I felt at ease and look forward to many more similar events in the future.’
• A thrill to have been there, so inspiring, keep up the amazing work.’
• Amazing effort! Sentences like “I cannot” look ridiculous to be said by anyone about anything after this event! Congratulations!’
• This event was truly inspirational. To hear the stories of the larynx group, accompanied by the beautiful words spoken by the children and then the great music really touched my heart. I think the work of all those involved should be applauded and supported. I hope in the future similar events can happen to raise awareness and get the needs of this condition more in the public eye.’
• Really interesting to see the work done and the progress made by the alaryngeal individuals, and learn more about the challenges they face and what can be done.’
• The speeches delivered by some of the participants were moving and thought-provoking. Understanding that the operation not only removes the voice box but also make the act of breathing so much harder gave me a new sense of respect and appreciation for what these people are going through. The courage and physical stamina they have shown in the face of their situation is a massive inspiration.’
• This was absolutely brilliant! I still feel blessed. Thank you for this experience.’
• A unique experience and approach to vocal development and requires lateral thinking and helps as a result and not only the feeling of rhythm but also understanding of the mechanics, aiding breathing control.’
• I was very impressed, particularly by the kids. The audience participation was a good idea—perhaps more of that in future.’
• This was an inspirational and informative event. The concept was so simple yet so uplifting. Thank you for the opportunity to hear patients, professionals, young people, and the public share in making amazing music together.’
• This charity gives patients such hope that where they are now, does not always have to be where they stay, they are not alone, and that they can achieve anything that they put their mind to. There is life after laryngectomy! And events like this will educate the public on these forgotten patients and their condition.’
• Fantastic!! Great community feel to the event, very entertaining and thought-provoking.’
• It was a wonderful and inspiring afternoon. What a fabulous project. I thought everyone involved especially the participants were amazing. It was also educational to hear details about the effects of not having a larynx. Profoundly moving experience.’

One comment in particular seemed to capture the ethos of this work and the importance of public engagement, stating ‘This event was an amazing celebration of why we need this type of public engagement. What a waste of time would it have been for all these wonderful people to practice beatboxing inside a hospital, in front of researchers!!! You just had to feel the energy in the room in order to understand how powerful this experience was for everybody, patients, children performers and audience. Many congratulations to all involved…’

## Discussion

This work involved a small group of laryngectomees and a novel approach to creative voice rehabilitation and development within a supportive and intimate environment. This reinforced the work of patients with voice professionals and encouraged an exploration of the patients’ voices through beatboxing. It also involved a final public performance in front of a broad audience. A major challenge for an activity that involves such an eclectic group of individuals is the vulnerability to absences and, unfortunately, three of our patient group became very ill and were unable to participate during the period in which the described workshops took place. For the same reason, we were unable to include someone with E speech in this group, although we had planned to do so.

Beatboxing is a vocal art that is popular amongst a younger audience, and to introduce it to an older patient group (mean age of 65 years) was initially, as expected, welcomed with some reservation. This is perhaps also reflected in the somewhat conservative scoring that some laryngectomees offered when asked to rate their experiences. However, the laryngectomy group responded well, overall, to the proposed exploration and exercises during the workshops. The recorded feedback was positive; the participants liked it, and it made them approach their voice in a different way, improving voice awareness and work on their pronunciation. All participants reported their engagement in this project has had beneficial effects on their phonation as well as their breathing and the control of support mechanisms.

In order to allow the patient group to have more time for exploring possible sounds systematically and to start developing their basic skills, we only introduced the young coparticipants in the last two sessions. The young participants picked up skills very quickly, with some exceeding the expected level of basic beatboxing techniques.

Although none of the young participants had been exposed to the laryngectomy voice or even heard of the condition before, they were intrigued and not intimidated, exhibiting an openness toward the patients. They were attentive during the introduction and the more detailed explanations about the layered impact after laryngectomy. During the workshops, both patients and participants interacted smoothly with each other. The young people asked questions freely and reacted in a collegial and respectful manner when the laryngectomy group was struggling or had to do a certain exercise at a slower pace. We included the young participants in introducing the small topics presented during the performance. This motivated them to interact with the patient group even more closely and also to prepare and perform background information searches. The societal awareness benefit to this project was not a prime objective, but such a secondary and tertiary gain for all participants anecdotally is of significant note.

The audience in the final public performance was very receptive to the interaction of the young participants with the laryngectomees, the background information and introductions, and the performances. This is also echoed in the written feedback offered, some of which is presented above.

The youth and their parents involved in the project not only found their participation to be rewarding but also reported enjoying being active participants in and contributors to public education and engagement; they reported learning a lot about the actual condition and also commented about being happy and proud to share their experience with the friends and family they had invited. Further, some offered to become more involved with charitable work. This approach of workshops, wherein the patient and target groups are interacting, seemed to be an effective way to explain and experience the impact of a condition. It certainly has inspired the research group and non-profit organization Shout at Cancer to identify an interest in the development of this model further and to organize workshops in different locations and/or with different target groups. This model is likely to be suitable for different types of conditions and these outcomes may be transferable to a wider patient and/or participant group; thus, we believe that it is worthy of further exploration. We note this is obviously something that requires future systematic scrutiny.

The patients seemed to have a stimulating effect on each other; as soon as one of them appeared to have mastered a certain technique or sound, the others found it easier to imitate. Even so, there was variability in the produced sounds and skills. We acknowledge that this was a very limited group of patients (*n* = 9 in total), but the positive outcomes and constructive interactions between patients suggest the need to further explore the use of workshops within a group context/environment. This experience suggests further research into the use of more experienced laryngectomy speakers in the voice recovery of patients who have recently had a laryngectomy would be worthwhile.

Furthermore, we discovered that these workshops demanded great concentration and that the exercises could often be tiring. In future workshops we will need to factor in additional time for resting, relaxation and recovery.

The alaryngeal participants were encouraged to allow some time for practice at home between workshops and in preparation for the final public performance. In reality, some of the participants reported they had not performed any alone. When asked why, they stated that not only are the group sessions a social activity that they enjoyed but also these sessions made it easier for them to perform these exercises. They reported that they did not feel incentivized to practice on their own, as it was the group experience that acted as the motivation catalyst. Meanwhile, others reflected that they did find time to do the rhythmical exercises because they were easily built into any type of music they are listening to, and they found themselves beatboxing along to many songs on numerous occasions.

To facilitate individuals’ exercising, we developed and made available relevant demonstration videos and pictures, based on the outcomes of the workshops. We aim to create more exercises at different levels of difficulty and interaction and integrate these in an online platform. The effectiveness of such a design is currently being assessed with further empirical research. This research will hopefully allow the team to offer support to individuals and groups and is hoped to also involve sessions for patients with varying levels of experience but also voice professionals that wish to support laryngectomees. Similar online services could help to improve accessibility to health care (in this case, voice recovery) in both developed and developing countries (Guo and Li, 2018).

## Conclusion

Laryngectomy affects a relatively small and scattered population with a higher incidence in the developing world, where there may be limited access to health care and support. The operation has a negative psychosocial impact, leading to high percentages of depression and social isolation among the affected population, where communication issues play an important role.

This public engagement project of beatboxing after laryngectomy used a series of workshops and a final public performance not only to increase awareness about throat cancer and its impact on people’s lives but also to explore the potential benefits of creative group participation of laryngectomees and to rehearse whether this has the potential to inform future research and practice.

We explored the implementation of beatboxing techniques into speech rehabilitation after laryngectomy. Both the research evidence from the literature and our somewhat limited empirical findings during the explorative workshops are supportive of the inclusion of beatboxing techniques in rehabilitation of the voice after laryngectomy. The overwhelmingly positive feedback that we have received suggests there is value in presenting how we structured our work and what methods we employed so that future research can build upon the significant effort invested in developing the essential materials, methods and media needed for this work. We adopted and translated some of our findings into educational materials and exercises supported by pictures and video tutorials. These materials are already being accessed by patients, carers and practitioners and are assets of a currently under development interactive technology platform that is intended to increase accessibility in voice recovery.

Clearly, there is a need for future systematic research within this context if a musico-therapeutic curriculum were to be established and/or if a battery were to be developed for the assessment of the effectiveness of such work as ‘intervention.’ Given the particular demographic and specific challenges that laryngectomees face on a daily basis, a future research design will need to be context sensitive, and we do not foresee a randomized control trial as applicable within the present paradigm.

Albeit a small number of participant laryngectomees, as a willing subset of what we believe to be the world’s only organized and systematically practicing and performing alaryngeal vocal ensemble, we found that beatboxing is an exciting, pluralistic, inclusive and very engaging way to introduce a safe synergistic environment within which laryngectomees engaged in creative activity using their novel vocal instruments. These activities were reported to offer greater benefits in allowing the laryngectomees to further develop their breathing as well as support-structures control whilst engaging in meaningful music-making.

## Data Availability Statement

The datasets generated for this study are available on request to the corresponding author.

## Ethics Statement

An ethics approval was not required as per the authors’ Institutions’ guidelines and national regulations. This was also confirmed by the joint Medical Research Council (MRC) and United Kingdom National Health System (NHS) Health Research Authority online ethics assessment tool (http://www.hra-decisiontools.org.uk/ethics/index.html). Nevertheless, written informed consent was obtained from all adult participants and from the parents/legal guardians of non-adult participants/performers.

## Author Contributions

TM and EH designed the research project, and conducted the workshops and public engagement performance. TM developed the materials and drafted the manuscript. EH designed the feedback instrument, collected and analyzed the feedback data, performed the further edits, and finalized the manuscript. All other authors offered feedback and comments for the completion of the final manuscript.

## Conflict of Interest

The authors declare that the research was conducted in the absence of any commercial or financial relationships that could be construed as a potential conflict of interest.

## Footnotes

1 http://www.mcld.co.uk/beatboxalphabet/

2 http://www.hra-decisiontools.org.uk/ethics/index.html

3 http://www.shoutatcancer.org/beatboxing

4 http://bit.ly/2Sl5Ye7

## Abbreviations

E: oesophageal
EL: electrolaryngeal
HNC: head and neck cancers
HPV: human papillomavirus
IPA: International Phonetic Alphabet
PE: pharyngoesophageal
SBN: Standard Beatbox Notation
SCC: squamous cell carcinoma
TE: tracheoesophageal
UCL: University College London
WHO: World Health Organization.
